# Three Cases of Serous Tubal Intraepithelial Lesions (STILs)

**DOI:** 10.7759/cureus.62895

**Published:** 2024-06-22

**Authors:** Mika Mizuno, Shinichi Togami, Kyoko Shirota, Ikumi Kitazono, Hiroaki Kobayashi

**Affiliations:** 1 Department of Obstetrics & Gynecology, Faculty of Medicine, Kagoshima University, Kagoshima, JPN; 2 Department of Gynecology, Hakuaikai Social Medical Corporation Sagara Hospital, Kagoshima, JPN; 3 Department of Pathology, Faculty of Medicine, Kagoshima University, Kagoshima , JPN

**Keywords:** see-fim protocol, serous tubal intraepithelial carcinoma, serous tubal intraepithelial lesion, high-grade serous carcinomas, p53 signature

## Abstract

Serous tubal intraepithelial carcinoma, serous tubal intraepithelial lesions (STILs), and the p53 signature are considered to be related to precursor lesions of high-grade serous carcinomas (HGSCs). However, the clinical significance and prognostic implications of these lesion types are currently unknown. We diagnosed three patients with STILs according to the morphological evaluation criteria and combined this with p53 and Ki-67 immunostaining. One patient had an HGSC of the ovary that was incidentally discovered at the time of ovarian cyst resection, and the HGSC in the other two patients was characterized after they underwent risk-reducing salpingo-oophorectomy. Herein, we present a report of three patients with STILs diagnosed based on clinical data and pathological findings, along with a review of the literature.

## Introduction

The theory that the origin of high-grade serous carcinomas (HGSCs) is the fallopian tube (FT) epithelium, with a serous tubal intraepithelial carcinoma (STIC) as a precursor lesion, is becoming well established [1-5]. Furthermore, p53 signature lesions and serous tubal intraepithelial lesions (STILs) are considered precursor lesions of STICs. Although there have been many histological and molecular investigations of precursor lesions in FTs with or without HGSCs, the clinical significance and prognosis of these lesions are still unknown. Additionally, comprehensive pathological examinations of the FTs are now routinely conducted in clinical practice, leading to the incidental discovery of STICs or STILs with increasing frequency. Herein, we report three cases of STILs along with a review of the literature.

## Case presentation

Case 1

**Figure 1:**
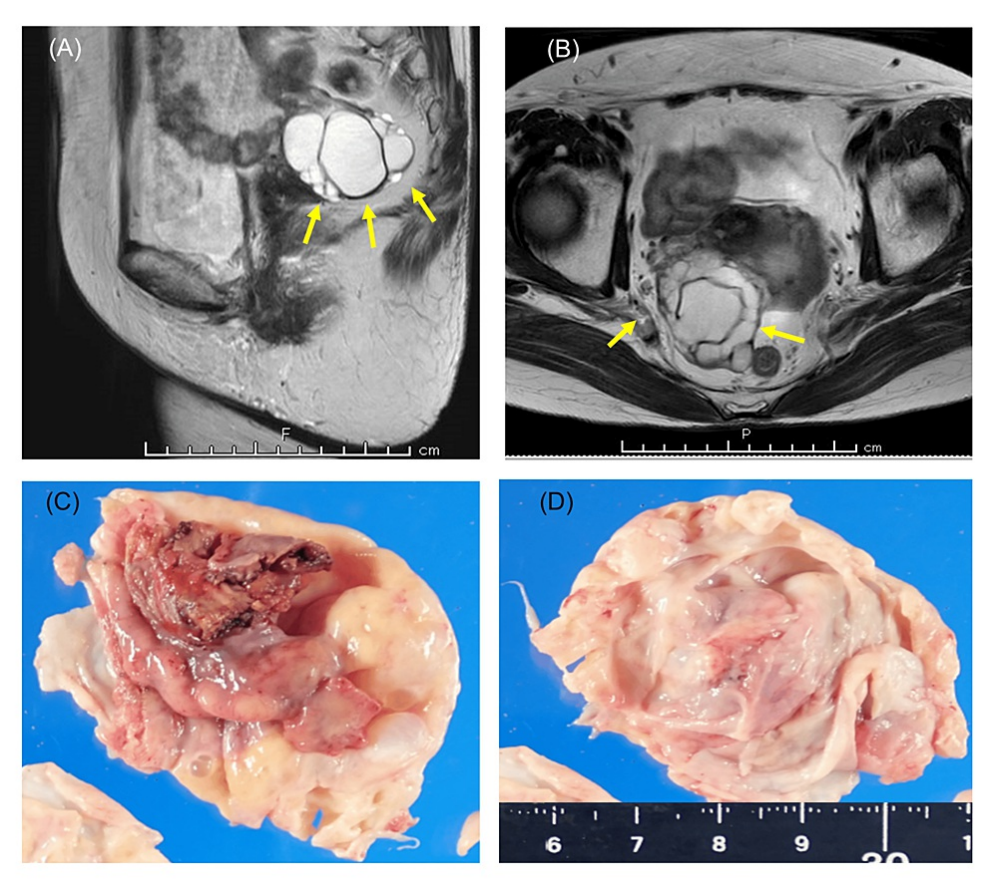
Preoperative images and gross findings of the surgical specimen for Case 1. (A and B): Pelvic magnetic resonance imaging (MRI) revealed multiple 6.5 cm long cysts without solid parts in the right ovary (arrows). There were no other abnormal findings. (C and D): Macroscopic image of the excised specimen (section of the right adnexal tumor).

A 68-year-old para-3 Japanese woman had been receiving immune checkpoint inhibitor therapy for three years for stage IV lung cancer with brain and mediastinal lymph node metastasis. This lung cancer was identified as non-small-cell lung cancer, specifically adenocarcinoma, characterized by the absence of EGFR gene mutation and ALK fusion gene, but with a programmed death-ligand 1 (PD-L1) tumor proportion score of 50% or higher, determined through immunohistochemistry (IHC). Therefore, pembrolizumab was administered at a dose of 200 mg per body every three weeks. This regimen markedly reduced the sizes of the primary lung cancer and its metastases. However, the latest computed tomography scan revealed that the size of the ovarian tumor had increased to 7 cm from 5 cm three years earlier. The pelvic magnetic resonance imaging (MRI) findings are shown in Figures 1A, 1B. Her blood test results, including her CA125 level, which was 6.8 U/mL, revealed no abnormalities. Both the cervical cytology and endometrial cytology were negative. She underwent laparoscopic bilateral salpingo-oophorectomy (BSO) on suspicion of a benign ovarian tumor. Neither ascites nor peritoneal dissemination was found. The resected specimen was placed in a bag and removed through the 12-mm trocar port. The right ovarian tumor was filled with serous fluid, and there was no visible solid component on the inside (Figures 1C, 1D). This tumor was considered a benign serous tumor according to rapid intraoperative pathological analysis. However, the final pathological diagnosis was stage IA (pT1aNXM0) ovarian carcinoma, which is an HGSC with a benign serous cystadenoma component constituting most of the tumor. Peritoneal washing cytology revealed some atypical epithelial cells. She prioritized continued pembrolizumab treatment for lung cancer and did not receive chemotherapy for this ovarian cancer. Approximately four years later, her lung cancer was stable, and her ovarian cancer had not recurred.

Case 2

A 49-year-old para-2 Japanese woman underwent laparoscopic risk-reducing BSO (RRSO) due to hereditary breast and ovarian cancer syndrome (HBOC). Her history was metachronous bilateral breast cancer with treatment six years earlier and two years earlier. One tumor was invasive lobular carcinoma that was hormone receptor-positive and human epidermal growth factor 2-positive, and the other was invasive ductal carcinoma that was hormone receptor-negative and human epidermal growth factor 2-positive. Both tumors had Ki-67 proliferation index values >30. She received chemotherapy and radiotherapy for her second breast cancer. There were no abnormal findings on diagnostic imaging or in the blood tests, including CA125 (7.0 U/mL). There were no macroscopic or washing cytology findings of malignant disease during surgery. Approximately 2.5 years later, she had no malignant tumors.

Case 3

A 59-year-old para-2 Japanese woman underwent laparoscopic hysterectomy and RRSO due to atypical endometrial hyperplasia and HBOC. She had a history of metachronous bilateral breast cancer treated 15 years earlier and two years earlier. She was treated with chemotherapy and radiation after the second breast cancer surgery. She also had a family history of breast cancer in a younger sister and three aunts. Approximately 1.5 years later, she had no disease recurrence and no malignant tumors.

Histological characteristics in three cases

The H&E staining and IHC analyses of the specimens surgically extracted for Case 1 and Case 2 were carried out. Immunostaining for Ki67 (clone MIB-1, Agilent Technologies Japan Ltd.), p53 (clone DO-7, Leica Biosystems Newcastle Ltd.), calretinin (clone CAL6, Ditto), and TTF-1 (clone SPT24, Ditto) was performed. We estimated the percentage of Ki-67-positive cells (i.e., the MIB-1-positive rate) in lesions with p53-positive areas.

Case 1

**Figure 2:**
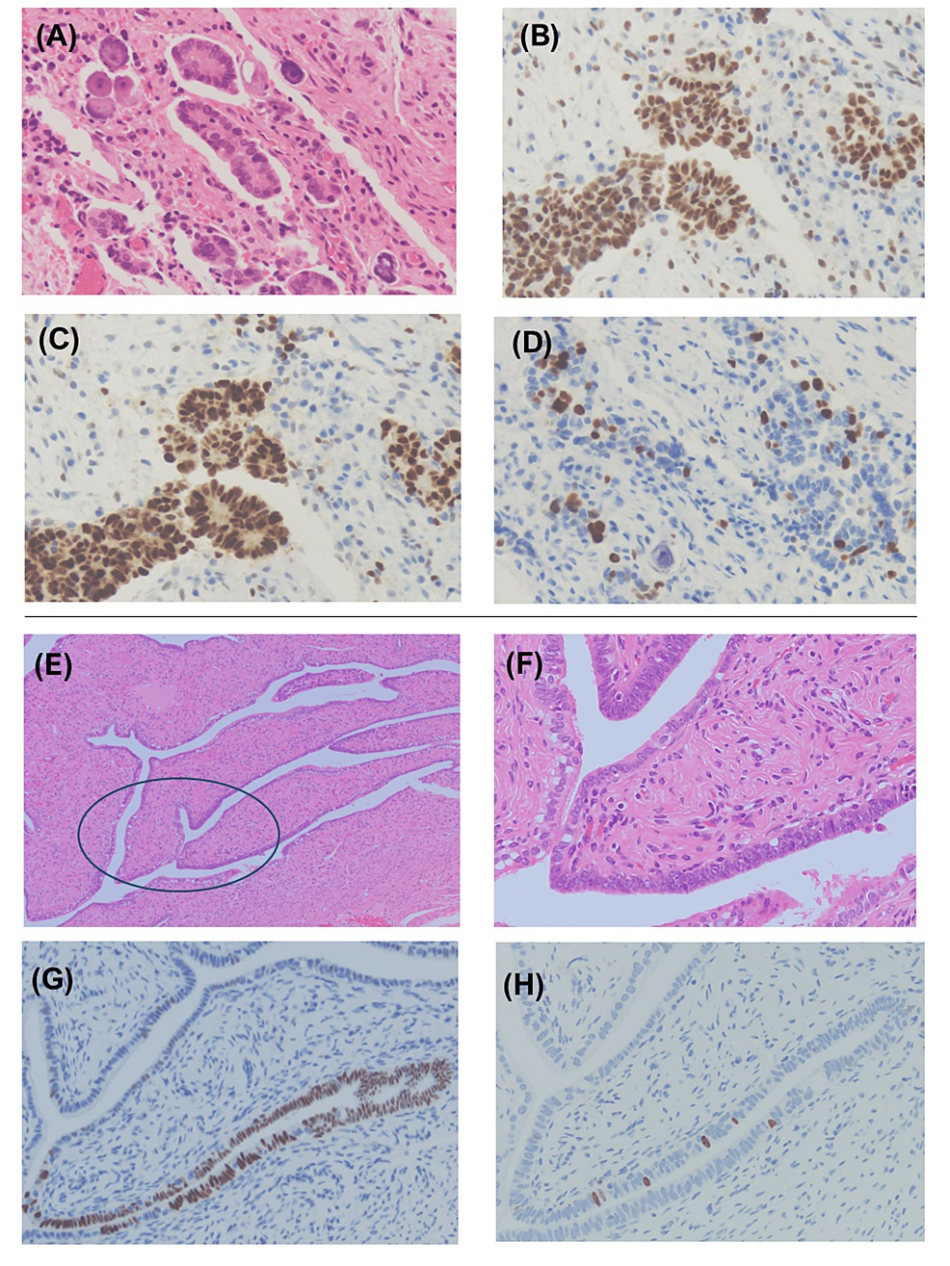
Histological and immunohistochemical features of the HGSC and the STIL in Case 1. Histological images of high-grade serous carcinoma (HGSC) of the right ovary and serous tubal intraepithelial lesion (STIL) of the right fallopian tube. (A-D) Pathological specimen of the right ovary. (E-H) Right fallopian tube. (A-E) H&E staining at ×200 magnification and (F) ×100 magnification; immunohistochemical staining (B and G): P53, (C) WT-1, and (D and H) Ki-67.

The right ovary had multilocular cysts, mostly serous cystadenomas with some papillary and glandular epithelium with atypical nuclei and loss of polarity. Lesions of the HGSC were found in a very narrow area of only one slice (Figure 2A). IHC of the epithelium revealed diffuse positivity for WT-1 (Figure 2C) and p53 (Figure 2B) and negativity for calretinin and TTF-1 (data not shown). The MIB-1-positive rate was 20% (Figure 2D). H&E staining of the right FT is shown in Figures 2E, 2F. Although there was mild atypia and a small portion of the epithelium was multilayered, ciliated cells with polarity showed a benign morphology. A strongly p53-positive region was found in a part of the right FT (Figure 2G), but the Ki-67 labeling index was less than 10% (Figure 2H). Thus, a STIC was not detected. Based on these findings, the patient was diagnosed with serous cystadenoma with a small HGSC component and a STIL in the right FT. Additional sections were obtained and reviewed again to obtain a more accurate diagnosis of the specimen. Finally, the STIC was not detected. We attempted to analyze the genomic profile of the specimens, but the HGSC sample was too small for examination.

Case 2

**Figure 3:**
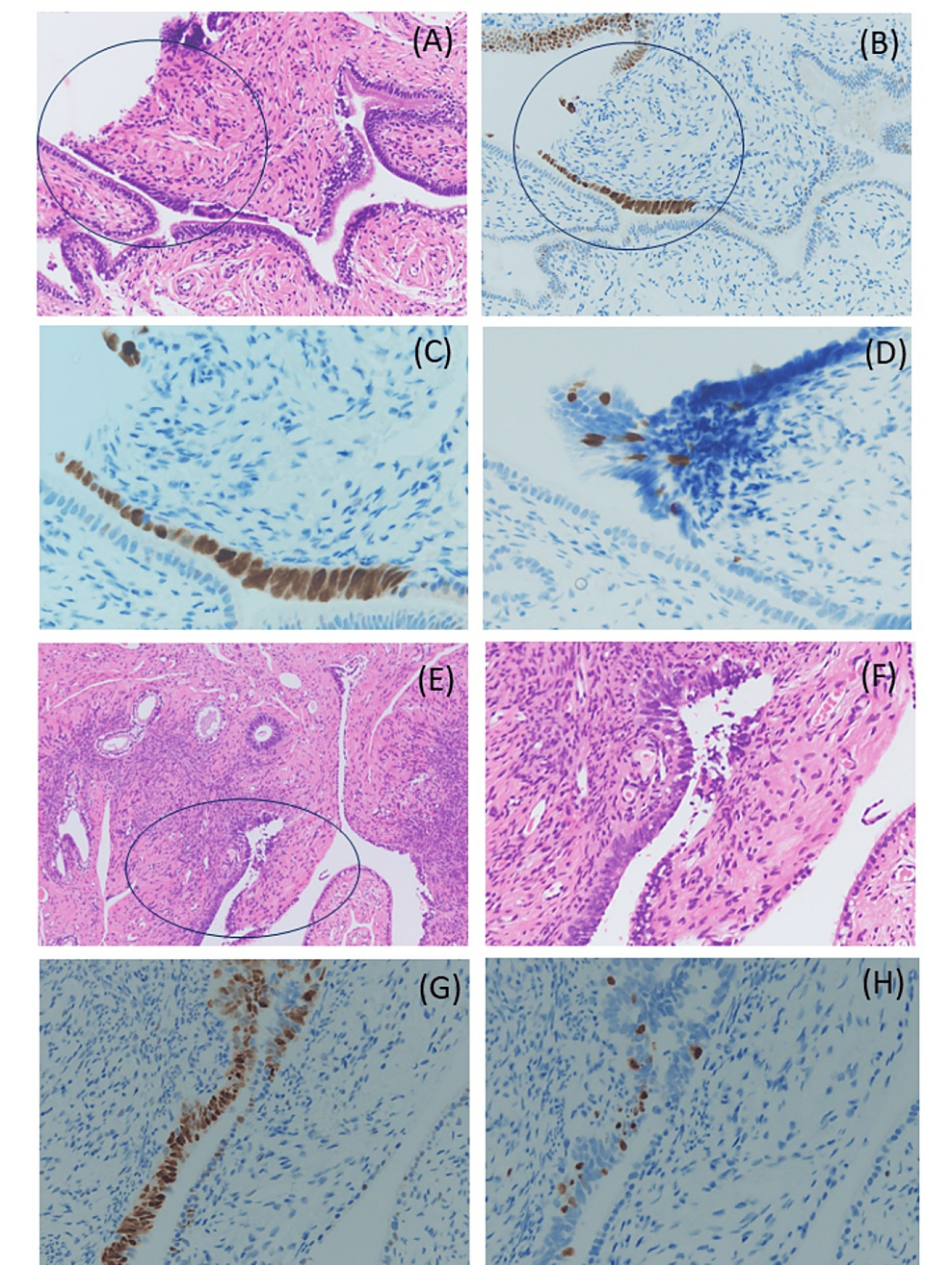
Histological and immunohistochemical features of the STIL on the left fallopian tube in Case 2. Two of the 15 sections of the left fallopian tube: (A-C) and (E-H), (A and E) H&E staining at ×100 magnification and (F) ×200 magnification; immunohistochemical staining (B, C, and G): P53 and (H) Ki-67.

Both ovaries/FTs were evaluated according to the Sectioning and Extensively Examining the FIMbriated End (SEE-FIM) protocol [6]. Two of the 15 sections of the left FT (Figures 3A-3D and Figures 3E-3H) were diagnosed as STILs. Ciliated cells with mild atypia that retained polarity showed a benign morphology similar to that in Case 1 (Figures 3A, 3E, 3F). p53-positive cells (Figures 3B, 3C, 3G) were detected, and the percentage of MIB-1-positive cells was less than approximately 10% (Figures 3G, 3H). STILs were found in only two areas of each tissue slice.

Case 3

Bilateral FTs were found to be locally positive for p53 regions based on the SEE-FIM protocol. Ciliated cells without atypia were found in p53-positive regions in the left FT, and ciliated cells with atypia and nuclear enlargement were found in the right FT. The MIB-1-positive rates were less than 5% and 8%, respectively, and each lesion was diagnosed with the p53 signature and STIL (no images or data shown).

## Discussion

Three patients with STILs, which were diagnosed according to the morphological evaluation criteria combined with p53 and Ki-67 immunostaining [7], are presented. One patient had an HGSC of the ovary that was incidentally detected at the time of ovarian cyst resection, and the other two patients had RRSO.

In 2007, Kindelberger et al. [5] first characterized the STIC, and according to the STIC theory, HGSCs in the FTs, ovaries, and peritoneum originate from the FTs [3,8]. This theory is based on the results of prospective/retrospective studies involving the examination of sliced whole FTs. The STIC is diagnosed based on morphological and immunohistochemical features, including a high nuclear-cytoplasmic ratio, the presence of pseudostratified depolarized nonciliated cells, and the ability to proliferate by replacing the FT epithelium that covers the existing FT structure. In the STIC, the nucleus has increased chromatin, is enlarged and polymorphic, and undergoes fission. Most patients exhibit TP53 abnormalities (overexpression or no expression [3]). The proliferation index, as assessed by the percentage of Ki-67-positive cells, is usually 10 or 15% or greater in STICs [5,7,9]. In addition, the rate of ER positivity was also high.

The process by which the STIC occurs is still unclear, but some reports suggest that the precursor state is assumed to include not only STIC but also the self-limiting p53 signature and STIL. Small lesions of nonciliated cells with Tp53 mutations are called p53 signatures in which DNA is damaged due to various factors [4,10]. STILs are considered lesions that may gradually develop following the transition from the p53 signature to STICs. STILs are diagnosed as nearly benign nonciliary cell tumors with abnormal p53 expression and a low percentage of Ki-67-positive cells [7].

A review of previous reports on STICs, STILs, and p53 signatures in the fallopian tubes is presented in Table 1, with some modifications from the original sources [11-14]. As shown in Table 1, a low percentage of STIC cases, along with STIL and p53 signatures, has been identified in both high-risk ovarian cancer populations [13] and groups with serous ovarian tumors [14]. Previously, when hysterectomy was performed for premenopausal women with benign tumors, the FTs with ovaries were preserved. Recently, bilateral salpingectomy (BS) for benign tumors has even become common due to the STIC theory. In 2022, a prospective study in which a detailed pathological examination of the FTs was performed on 273 patients who underwent hysterectomy and BS for benign tumors was reported [12]. p53 signatures were detected in approximately 80% of the FT samples, and the STIL and STIC were detected in 3.3% and 0%, respectively, of the samples. Although the impact of BS on prognosis is unknown, these data cannot be overlooked. Furthermore, in the most recent report [11], among 13,936 patients who underwent surgery for major benign conditions, 91 patients with incidental p53 signatures unrelated to ovarian cancer were identified. This group of 91 patients included 29 patients with uterine cancer or complex atypical endometrial hyperplasia. Among these, 30 patients (33.0%) had a personal history of nonovarian cancer, including cervical (1), breast (6), or other cancers, and 20 patients (21.9%) had concurrent primary uterine carcinoma. Regarding family history, 19 patients (20.9%) reported a family history of ovarian cancer, 28 patients (30.8%) had a family history of breast cancer, and seven patients (7.7%) had a family history of uterine cancer. At least in gynecological surgery for women who do not wish to become pregnant, BS is not disadvantageous; therefore, it is thought that tubal resection should be actively considered.

**Table 1:** Detection rates of the STIC, STIL, and the p53 signature. STIC: serous tubal intraepithelial carcinoma, STIL: serous tubal intraepithelial lesion, HBOC: hereditary breast and ovarian cancer, PAX 8: paired box 8

Author (Year)	Study Content	N	Main Findings	Frequency of Lesions
Munakata & Yamamoto (2015) [14]	Incidence of STIC in patients with serous ovarian tumors	55	Found STIC exclusively in serous carcinoma cases and indicated PAX8 expression as significantly associated with serous tumors.	p53 signatures: 1.8% STIL: 5.5%, STIC: 9.1%
Visvanathan et al. (2018) [13]	Multicenter study on fallopian tube lesions in patients with high risk of ovarian cancer	479	Many lesions in fallopian tube were found in BRCA1/2 mutation carriers.	p53 signatures: 27%, STIL: 1.3%, STIC: 6.3%
Tchartchian et al. (2022) [12]	Prospective study of prophylactic salpingectomy for benign conditions	273	Prophylactic salpingectomy may reduce the risk of ovarian cancer.	p53 signatures:80%, STIL: 3.3%, STIC: 0%
MacArthur et al. (2024) [11]	Clinical outcomes following identification of an incidental p53 signature in the fallopian tube	13,936	Among 91 patients with p53 signatures, 5 (5.5%) were diagnosed with non-ovarian malignancies, and no ovarian or peritoneal cancers were observed during follow-up.	p53 signatures: 0.9%, 0.6% without ovarian cancer, STIC, or HBOC

On the other hand, there are also questions about whether all STICs develop into invasive cancer and whether STILs are involved [13,15]. The genomic landscape of the FT precursor lesions and HGSC was analyzed using whole-exome sequencing and amplicon sequencing in women with/without ovarian carcinoma. The results in women with HGSCs showed that while there are common TP53 mutations, there are nonidentical mutations between precursor lesions and carcinoma. The results suggested not only stepwise tumor progression through precursor lesions but also the diverse clonal origins of tubal precursor lesions during oncogenic transformation. Additionally, in the aforementioned study of 91 patients diagnosed with p53 signatures, seven patients (7.7%) underwent additional surgery following the p53 signature diagnosis, and 19 patients (20.9%) retained their ovaries [12]. During the mean follow-up period of 27 months, there were no reports of associated ovarian cancer.

Another problem is the standardization of testing accuracy and uniformity among facilities. The 5th edition of the WHO Classification [16] suggests identifying the primary lesion of HGSCs using the SEE-FIM protocol for identifying STICs or HGSCs present in either the FT or ovary, similar to RRSO. However, there are gaps in the methods and diagnostic capacities of each medical institution. At present, differences in the primary tumor of HGSCs do not affect the prognosis, but the diagnostic accuracy may affect the outcome not only for RRSO but also for surgery for benign tumors suspected of being HBOC.

## Conclusions

We identified three cases of STILs: two cases were identified by RRSO, and one case, which included a small HGSC component, was incidentally found during laparoscopic surgery for a serous cystic tumor. The outcomes for these three cases have been favorable. The clinical significance of STILs and p53 signature lesions is unclear, and research on the frequency and prognosis of STILs and STICs in long-term, large-scale trials is needed.
